# Phenotypic Dissection of a *Plasmodium*-Refractory Strain of Malaria Vector *Anopheles stephensi*: The Reduced Susceptibility to *P. berghei* and *P. yoelii*

**DOI:** 10.1371/journal.pone.0063753

**Published:** 2013-05-23

**Authors:** Naoaki Shinzawa, Tomoko Ishino, Mayumi Tachibana, Takafumi Tsuboi, Motomi Torii

**Affiliations:** 1 Department of Molecular Parasitology, Ehime University Graduate School of Medicine, Toon, Ehime, Japan; 2 Cell-free Science and Technology Research Center, Ehime University, Matsuyama, Ehime, Japan; Metabiota, United States of America

## Abstract

Anopheline mosquitoes are the major vectors of human malaria. Parasite-mosquito interactions are a critical aspect of disease transmission and a potential target for malaria control. Current investigations into parasite-mosquito interactions frequently assume that genetically resistant and susceptible mosquitoes exist in nature. Therefore, comparisons between the *Plasmodium* susceptibility profiles of different mosquito species may contribute to a better understanding of vectorial capacity. *Anopheles stephensi* is an important malaria vector in central and southern Asia and is widely used as a laboratory model of parasite transmission due to its high susceptibility to *Plasmodium* infection. In the present study, we identified a rodent malaria-refractory strain of *A. stephensi mysorensis* (Ehime) by comparative study of infection susceptibility. A very low number of oocysts develop in Ehime mosquitoes infected with *P. berghei* and *P. yoelii*, as determined by evaluation of developed oocysts on the basal lamina. A stage-specific study revealed that this reduced susceptibility was due to the impaired formation of ookinetes of both *Plasmodium* species in the midgut lumen and incomplete crossing of the midgut epithelium. There were no apparent abnormalities in the exflagellation of male parasites in the ingested blood or the maturation of oocysts after the rounding up of the ookinetes. Overall, these results suggest that invasive-stage parasites are eliminated in both the midgut lumen and epithelium in Ehime mosquitoes by strain-specific factors that remain unknown. The refractory strain newly identified in this report would be an excellent study system for investigations into novel parasite-mosquito interactions in the mosquito midgut.

## Introduction

Malaria persists today as the most widespread and devastating protozoan disease of humans. It is one of the major causes of mortality in children under 5 years of age in sub-Saharan Africa. The causative agents of malaria are protozoan parasites of the genus *Plasmodium*, which are transmitted to humans when female *Anopheles* mosquitoes feed on blood. The parasite obligatorily undergoes the sexual stages of its life cycle in the mosquito. Shortly after the ingestion of an infected blood meal, male and female gametocytes develop into gametes in the mosquito midgut. When male gametocytes undergo endomitotic divisions, axonemes are assembled, each of which becomes the motile backbone of a flagella-like microgamete. In a spectacular process termed exflagellation, microgametes are finally expelled from the residual body of the male gametocyte. After exflagellation and fertilization, the newly formed zygotes transform into motile ookinetes that invade and cross the midgut epithelium between 24 and 48 h post-infection. Once they reach the basal lamina, the ookinetes undergo arrest and develop into protected capsules, called oocysts. Several mitotic divisions within each oocyst give rise to thousands of sporozoites, which are released into the hemocoel following oocyst rupture, approximately 2 weeks after infection. The sporozoites are carried to the salivary glands and traverse the gland epithelium into the salivary gland lumen, where they further mature before being transferred to a new human host.

Of the 400 anopheline species worldwide, only 40 are important malaria vectors. One of these, *Anopheles gambiae*, may transmit *Plasmodium falciparum*, the parasite responsible for the cerebral form of malaria in much of sub-Saharan Africa. The L3–5 *Plasmodium*-refractory strain of *A. gambiae* was isolated from the G3 colony using genetic selection for resistance to simian malaria *P. cynomolgi* infection, and the 4Arr susceptible strain was selected from a combination of colonies from Liberia and Kenya [1]–[3]. A subsequent study demonstrated that polymorphisms of a single gene encoding antiparasitic thioester-containing protein 1 (TEP1) explain a substantial part of the variability in parasite susceptibility [4]. The genetic loci related to *P. falciparum* infection intensity have been identified through quantitative trait loci analysis of isofemale colonies [5], [6]. Both field and laboratory inter-species studies of *Plasmodium* refractoriness have been conducted with various combinations of *Anopheles* and *Plasmodium* [7]–[9]. Comparisons between the *Plasmodium* susceptibility of different anopheline mosquitoes represent a fundamental contribution to the understanding of parasite-mosquito interactions and suggest that genetic diversity raises vectorial capacity, based on intricate dyadic interactions.

*A. stephensi* is a sub-tropical species that is distributed throughout the Middle East and South Asia. It is a major vector of malaria in urban areas in India, accounting for apporoximately 12% of malaria cases annually, and is also an important malaria vector in Pakistan and Iran [10], [11]. *A. stephensi* has a broad and moderately high susceptibility to infection with various malaria parasite species and strains, regardless of where the mosquitoes originated [12]–[14]. On the basis of egg morphological characters, three biological forms of this mosquito species have been reported: *A. stephensi stephensi* (type), intermediate, and *A. stephensi mysorensis* [10], [15]. However the *Plasmodium* susceptibility of these biological forms has not yet been compared.

Genetic selection of *A. stephensi* Liston was performed using *P. falciparum* infection, and the susceptible line SDA500 was isolated [16], [17]. This highly susceptible line is widely used for phenotypic studies of parasite development [18], [19]. In the current study, we compared the susceptibility of two strains of *A. stephensi* to infection with rodent malaria parasites: the SDA500 intermediate form and the Ehime *mysorensis* form. The Ehime *mysorensis* mosquitoes were from a closed colony that has been reared at Ehime University for over 15 years. We found that the Ehime strain displayed high refractoriness to infection by the rodent malaria parasites *P. berghei* and *P. yoelii*, which are highly infectious to SDA500. Using stage-specific analysis, we identified incomplete differentiation of ookinetes in the midgut lumen and impaired crossing of the midgut epithelium as the reasons for the low number of mature oocysts in these mosquitoes. This explains the *Plasmodium* refractoriness of Ehime mosquitoes, and the genetic background of these strains governs this variation in susceptibility. Our observations suggest that the Ehime strain can be a used as a new tool to study parasite-vector interactions, particularly the genetic factors that limit the crossing of the midgut epithelium by parasites and parasite development in the midgut lumen.

## Results

### The Ehime Strain is a *Plasmodium*-refractory *A. stephensi*

**Figure 1:**
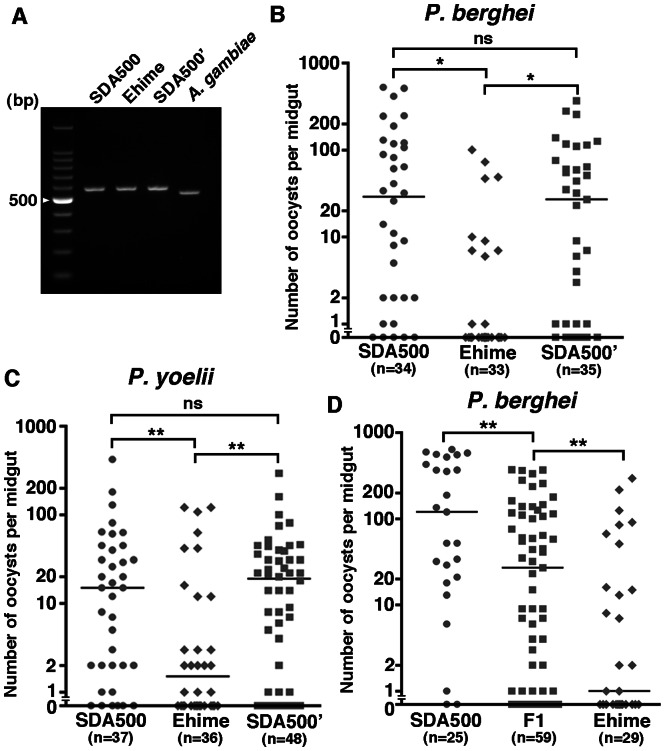
*A. stephensi* Ehime exhibits infection refractoriness to rodent malaria parasites. (A) Agarose gel electrophoresis after PCR with ITS2 region-specific primers for the genomic DNA of representative mosquitoes: SDA500, from Kagawa University; SDA500’, from Mie University. (B–D) Persistence of malaria oocysts on the basal lamina of infected mosquitoes. Mosquitoes were infected with fluorescent parasites and were dissected after maturation of oocysts. All dissected mosquitoes were blood-fed using the same mouse in each experiment. The number of oocysts in each midgut was counted. The dots represent the number of oocysts present on individual midguts and the median number of oocysts is indicated by the horizontal line. The total number of mosquitoes blood-fed is indicated under each group’s name. (B and C) Persistence of malaria oocysts on the basal lamina of two strains of SDA500 and Ehime mosquitoes infected with fluorescent parasites. Infection phenotypes were confirmed in three independent experiments using a different infected mouse. (D) Persistence of malaria oocysts on the basal lamina of F_1_ progeny from a reciprocal cross of Ehime and SDA500 mosquitoes and their parental lines. Infection phenotypes were confirmed in two independent experiments using a different infected mouse. *p<0.0001, **p<0.01 (Mann-Whitney test), ns: not significant.

We first confirmed the anopheline species of Ehime-reared mosquitoes (Ehime) by sequencing analysis of internal transcribed spacer 2 (ITS2) [20]. Using a pair of PCR primers that anneal to the 5.8S and 28S coding regions of all species so far examined within the genus *Anopheles*, the intervening ITS2 sequence was amplified from Ehime, two *A. stephensi* SDA500 referential lines (SDA500 and SDA500’) that were reared in different laboratories, and *A. gambiae* G3. Following electrophoresis of the PCR products through a 2% agarose gel (Figure 1A), a single band migrating at approximately 590 bp was visible for Ehime and *A. stephensi* SDA500. This band was clearly distinguishable from the corresponding PCR products from *A. gambiae* (∼550 bp). Sequences obtained from Ehime and SDA500 matched completely with those in GenBank (Accession number: AY157316 [21]) (Figure S1). Next, we focused on the biological forms of *A. stephensi*. In order to identify the biological form of Ehime, the ridges on the egg float were counted, revealing that SDA500 and Ehime were the intermediate and *mysorensis* forms, respectively (Table 1). Therefore, the Ehime mosquito is a strain of *A. stephensi*, but is a different biological form to the SDA500 strain.

**Table 1:** Number of ridges on egg float of SDA500, Ehime and *A. stephensi* caught wild in Iran.

Strains (biological form)	No. of ridges (Mean ± SD)	No. of eggs examined	Reference
SDA500 (Intermediate)	15.14±1.56	50	this study
Ehime (*mysorensis*)	13.40±1.22	50	this study
Type	18.7±1.70	513	[10]
Intermediate	15.4±0.89	129	[10]
*mysorensis*	13.6±1.30	81	[10]

To investigate the vectorial capacity of the Ehime strain, mosquitoes were infected with rodent *Plasmodium*. The number of oocysts that formed on the basement membrane after infection indicated that the Ehime strain is a refractory strain for *P. berghei* infection. In contrast, SDA500, which is known to be a highly susceptible line [16], [17], contained many more developed oocysts than the Ehime strain (Figure 1B and Figure S2). The prevalence of *P. berghei* oocysts was significantly lower in the Ehime strain than in the SDA500 or SDA500’ strain (Table 2 and Table S1). The Ehime strain showed similar refractoriness to infection by another rodent malaria parasite, *P. yoelii* (Figure 1C and Figure S3). The prevalence of *P. yoelii* oocysts was also significantly lower in the Ehime strain than in the SDA500 or SDA500’ strain (Table 2 and Table S1). To explore whether Ehime refractoriness depended on genetic background, we set up reciprocal crosses of refractory Ehime and susceptible SDA500 strains. F_1_ progeny and mosquitoes from their parental lines were infected with *P. berghei* from a single mouse. The resulting F_1_ mosquitoes exhibited an intermediate phenotype (Figure 1D, Table 2, Figure S4, and Table S1). These infection studies suggest that, based on the assessment of oocyst formation alone, Ehime mosquitoes have high refractoriness to *Plasmodium* infection that is controlled by complicated genetic differences between biological forms.

**Table 2:** Decreased prevalence of *P. berghei* and *P. yoelii* oocysts in Ehime *A. stephensi.*

Experiments	Parasite	Mosquitostrain	n	Prevalence(%)^a^	p-value^b^
Mouse 1	*P. berghei*	SDA500	34	85.3	<0.001^c^
(Figure 1B)		Ehime	33	33.3	–
		SDA500’	35	80.0	<0.001^c^
Mouse 2	*P. yoelii*	SDA500	37	83.8	<0.05^c^
(Figure 1C)		Ehime	36	58.3	–
		SDA500’	48	81.3	<0.05^b^
Mouse 3	*P. berghei*	SDA500	25	92.0	<0.01^c^
(Figure 1D)		F_1_	59	83.1	ns^d^, <0.05^c^
		Ehime	29	55.2	-

a The oocyst prevalence was calculated by dividing the the number of oocyst-positive mosquitoes by the total number of blood-fed mosquitoes.

b p-value was calculated by chi-square analysis.

c compared with oocyst prevalence in Ehime.

d compared with oocyst prevalence in SDA500.

### Exflagellation of Male Gametes is Normal in the Ehime Strain

**Figure 2:**
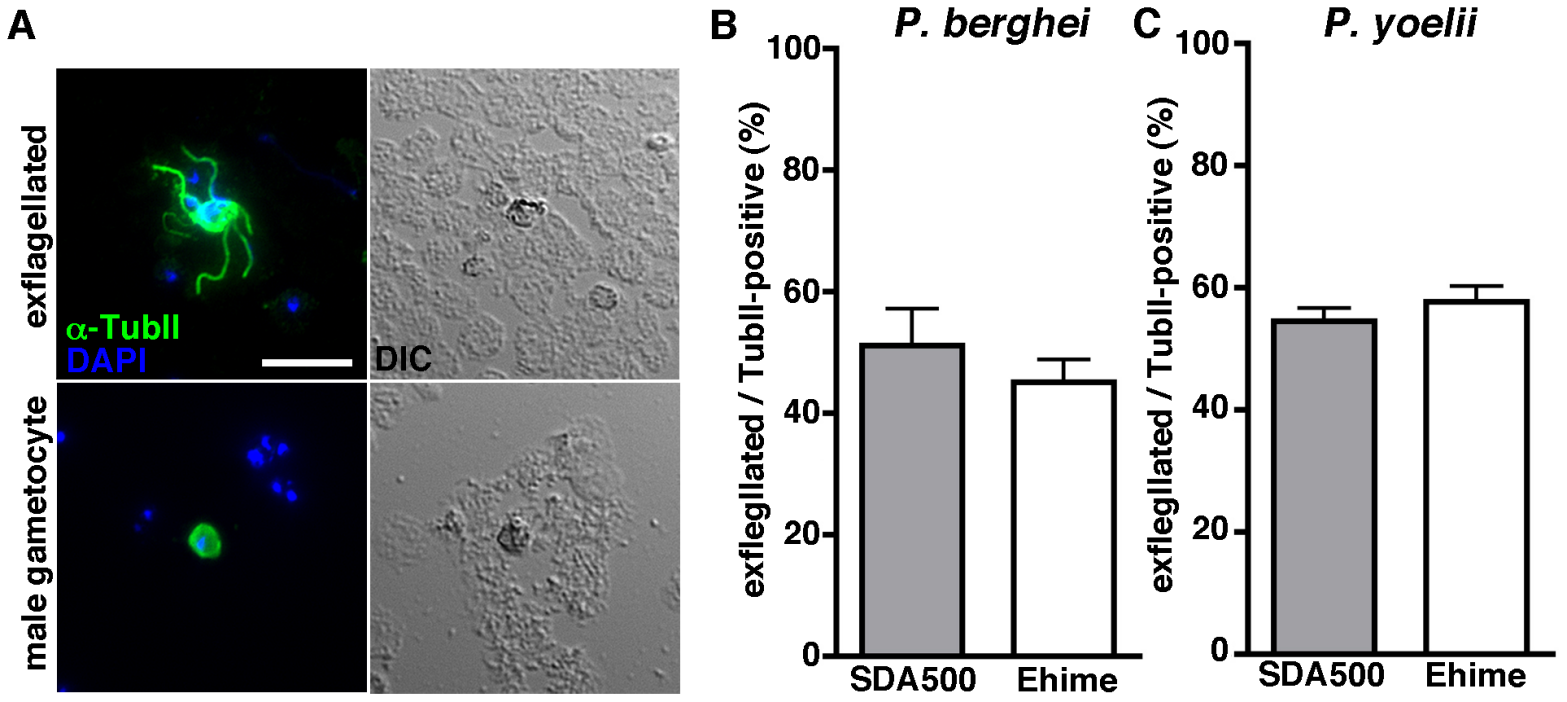
Normal exflagellation of male parasites is observed in the midgut of Ehime mosquitoes. (A) Anti-TubII antiserum stains both male gametes and male gametocytes. Mosquitoes were infected with Pb-GFP, dissected 10–15 min after feeding, and the plated blood meal was stained with anti-TubII antiserum (Green) and DAPI (Blue) following acetone fixation. Anti-TubII antiserum cross-reacts with the paralogue of *P. berghei*, and was used as a positive marker for both male gametocytes and male gametes. Both male gametes with exflagellation (upper) and immature male gametocytes (lower) were observed. Scale bar, 10 µm. (B and C) Frequency distribution of male gametes with exflagellation in the midgut lumen of the SDA500 and Ehime strains with *P. berghei* infection (B) or *P. yoelii* infection (C). SDA500 (left) and Ehime (right) mosquitoes were infected with Pb-GFP or Py-RFP. Mosquitoes were dissected 10–15 min after feeding, the spotted blood meal was stained with anti-TubII antiserum, and the average proportion of 100 male gametes with exflagellation in five mosquitoes was estimated. Error bars represent standard deviation of four independent assays using a different infected mouse. Not significant, for Pb-exflagelaltion rate in SDA500 *vs*. Ehime (Unpaired t-test). Not significant, for Py-exflagelaltion rate in SDA500 *vs*. Ehime (Unpaired t-test).

To determine the parasitic stage at which the development of gametes is affected, we first assessed whether male parasites could activate in the ingested blood in the midgut lumen of the Ehime strain. To verify the exflagellation of male gametes, we developed a specific antibody for male-specific alpha-tubulin II [22]. A mouse antiserum for alpha-tubulin II (anti-TubII) specifically labeled male *P. berghei* and *P. yoelii* parasites. Therefore, the shape of the stains on slides of ingesta taken from the midgut of mosquitoes helped us to detect emerged male gametes of *P. berghei* after exflagellation (Figure 2A). Using this assay, we observed similar levels of male *P. berghei* gamete emergence in the midgut of the Ehime and SDA500 strains within 15 min after infected blood-feeding, thus demonstrating normal development of sexual-stage parasites in the refractory mosquitoes (Figure 2B). The same result was obtained with *P. yoelii* (Figure 2C). These observations imply that any differences in the synthetic pathways of the mosquito-derived xanthurenic acids (XA), which induce exflagellation in the midgut lumen, of the examined strains are not significant. Given that XA are produced via the tryptophan pathway that is linked to the eye color of dipteran insects [19], [23], the similarity of XA-synthetic pathways in the examined strains is supported by the same, normally pigmented, eye color of the Ehime and SDA500 strains (data not shown).

### Impaired Invasive-stage Parasites are Responsible for the *Plasmodium-*refractoriness in Ehime Mosquitoes

**Figure 3:**
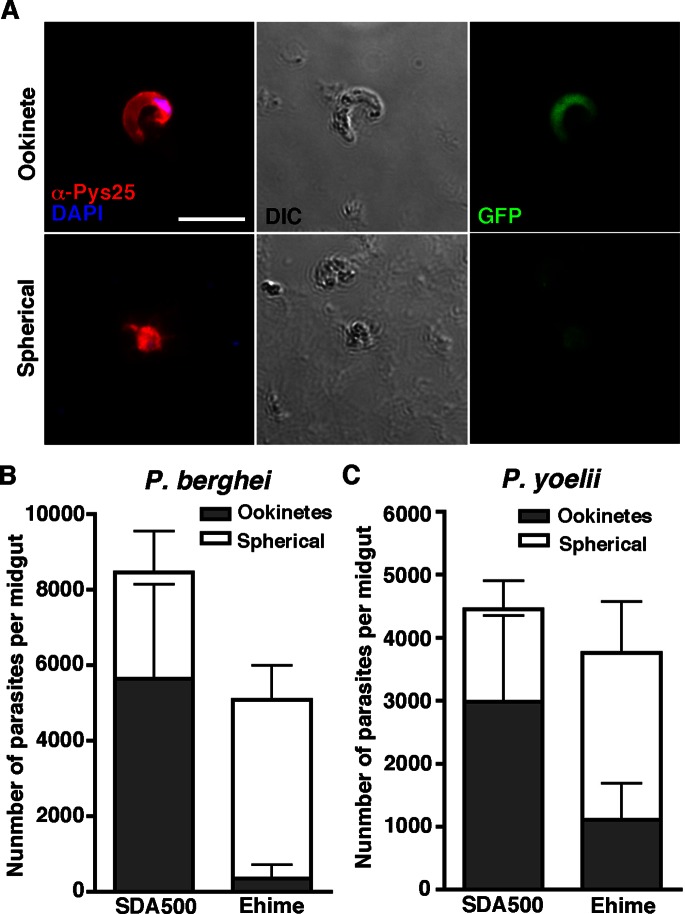
Incomplete differentiation of malaria ookinetes is observed in the midgut of Ehime mosquitoes. (A) Anti-Pys25 antibody distinguishes ookinetes in ingested blood. Mosquitoes were infected with Pb-GFP, dissected 18 h after feeding, and the plated blood meal was stained with anti-Pys25 (Red) and DAPI (Blue) following paraformaldehyde fixation. Pys25 is a positive marker for the surface of zygotes and ookinetes, and its antibody cross-reacts with Pbs25. Although normally developed ookinetes are GFP-positive and Pys25 signals appear on their surfaces (upper), spherical parasites display impaired GFP fluorescence and dispersed Pys25 signals (lower). Scale bar, 10 µm. (B and C) Frequency distribution of degenerated parasites and ookinetes in the midgut lumen of SDA500 and Ehime strains with *P. berghei* infection (B) or *P. yoelii* infection (C). Ehime and SDA500 mosquitoes were infected with Pb-GFP or Py-RFP and dissected. Spotted blood meals were stained with anti-Pys25, and the number of spherical parasites (white) and ookinetes (gray) were estimated. Error bars represent standard deviation; n = 20. p<0.001, for Pb-ookinetes in SDA500 *vs.* Ehime (Mann-Whitney test), p<0.01, for Py-ookinetes in SDA500 *vs.* Ehime (Mann-Whitney test). Infection phenotypes for *P. berghei* were confirmed in three independent experiments using a different infected mouse. Infection phenotypes for *P.yoelii* were confirmed in two independent experiments using a different infected mouse.

We assessed whether ookinete formation was inhibited prior to invasion in refractory mosquitoes using a staining surface marker, P25 (*P. berghei*: Pbs25, *P. yoelii*: Pys25). The monoclonal antibody for Pys25 cross-reacts with Pbs25 [24]. The staining analysis identified normally developing *P. berghei* ookinetes and spherical parasites in luminal parts 18 h post blood-feeding (Figure 3A). Spherical parasites with reduced fluorescence were also detected and may be parasites that were degenerating or dying during ookinete transformation (Figure 3A, lower panel). Counting the ookinetes and spherical parasites indicated a low number of *P. berghei* ookinetes in the ingested blood in the midgut lumen of Ehime mosquitoes (Figure 3B and Figure S5). Infection with *P. yoelii* also resulted in impaired ookinete formation in Ehime mosquito midguts (Figure 3C and Figure S5). This indicates that differentiation of zygotes into ookinetes is inhibited in the Ehime midgut.

**Figure 4:**
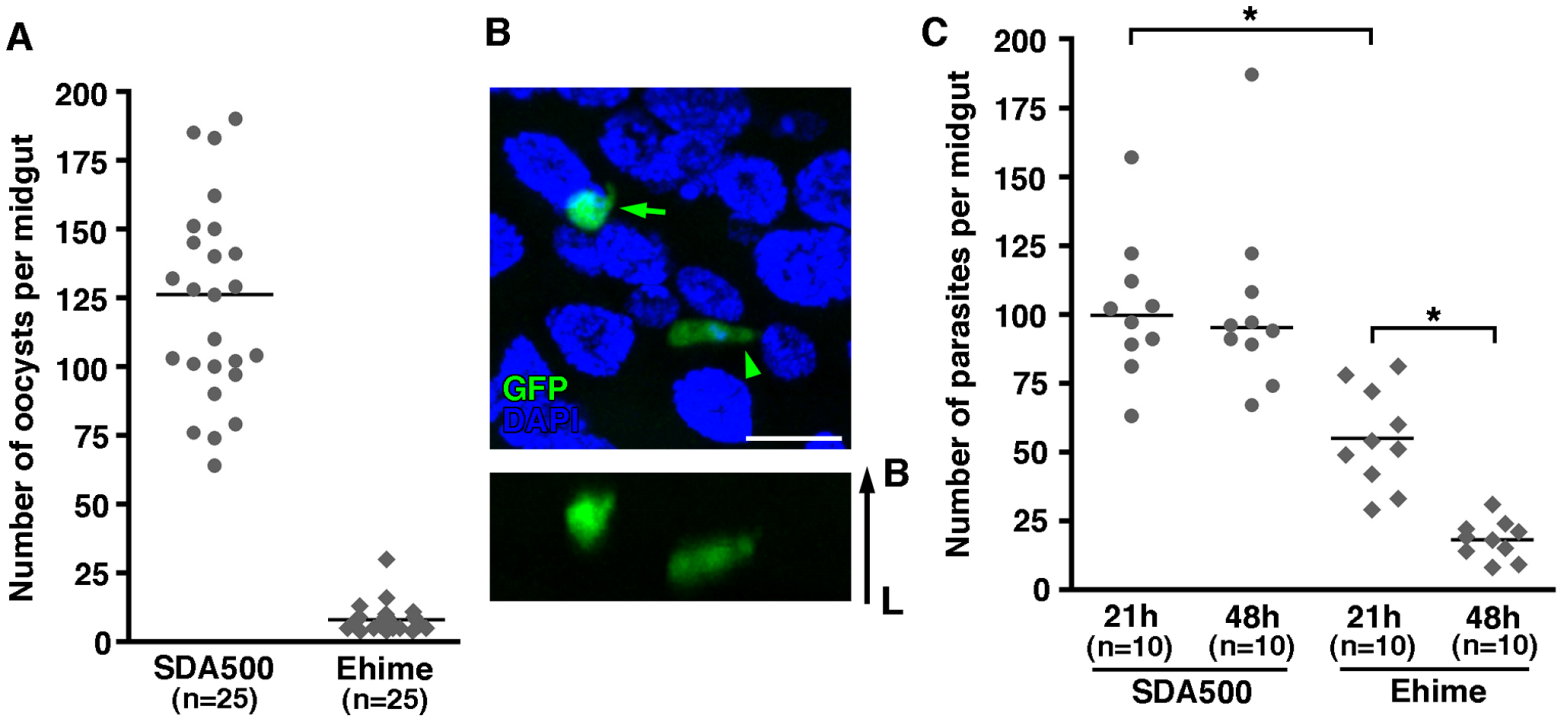
Impaired crossing of cultivated ookinetes in the midgut epithelium of the Ehime strain. (A) Persistence of malaria oocysts in the basal lamina of SDA500 and Ehime mosquitoes after feeding on ookinetes. SDA500 and Ehime mosquitoes were fed cultivated Pb-GFP ookinetes and dissected 5 days after feeding. The number of oocysts in each midgut was counted. The dots represent the number of oocysts present on individual midguts and the median number of oocysts is indicated by the horizontal line. The total number of mosquitoes blood-fed is indicated under each group’s name. p<0.0001 for oocysts in SDA500 *vs.* oocysts in Ehime (Mann-Whitney test). Infection phenotypes were confirmed in three independent experiments using different cultivated ookinetes. (B) Confocal microscopic image of a young oocyst (arrow) and an invading ookinete (arrowhead) observed in an infected midgut. At 21 h after Pb-GFP ookinete feeding, infected midguts were dissected, and nuclei were stained with DAPI (blue) after fixation. A vertical arrow indicates the direction of the midgut epithelium: start, lumen (L); end, and basal lamina (B). (C) Persistence of malaria parasites in the midgut of SDA500 and Ehime after feeding on ookinetes. Infected midguts were dissected at 21 and 48 h post feeding (hpf). The number at 21 hpf includes both invading ookinetes and young oocysts, and the number at 48 hpf indicates early oocysts. The total number of mosquitoes blood-fed is indicated under each group’s name. *p<0.0001 (Mann-Whitney test). Infection phenotypes were confirmed in two independent experiments using different cultivated ookinetes.

We next analyzed ookinete traversal of the mosquito midgut epithelium because a mosquito complement-like factor mediates the midgut defense system [25], [26]. To test ookinete traversal of the midgut, mosquitoes were fed *in vitro*-cultivated *P. berghei* ookinetes. There were significantly fewer oocysts found on the hemolymph side of the midgut epithelium in the Ehime strain than were found on the hemolymph side of the midgut epithelium of SDA500 mosquitoes (Figure 4A and Figure S6). Further observation of both invading ookinetes and newly developed oocysts in refractory mosquitoes revealed that the number of invading *P. berghei* ookinetes was low, and that many parasites were lost before early oocyst formation (Figure 4, B and C, and Figure S7). Therefore, in Ehime mosquitoes, ookinete formation is impaired in the midgut lumen and developed ookinetes are eliminated during crossing of the midgut, suggesting that the refractoriness demonstrated by the Ehime strain strongly affects invasive-stage parasites.

### Development of Oocysts is Normal in Ehime Mosquitoes

**Figure 5:**
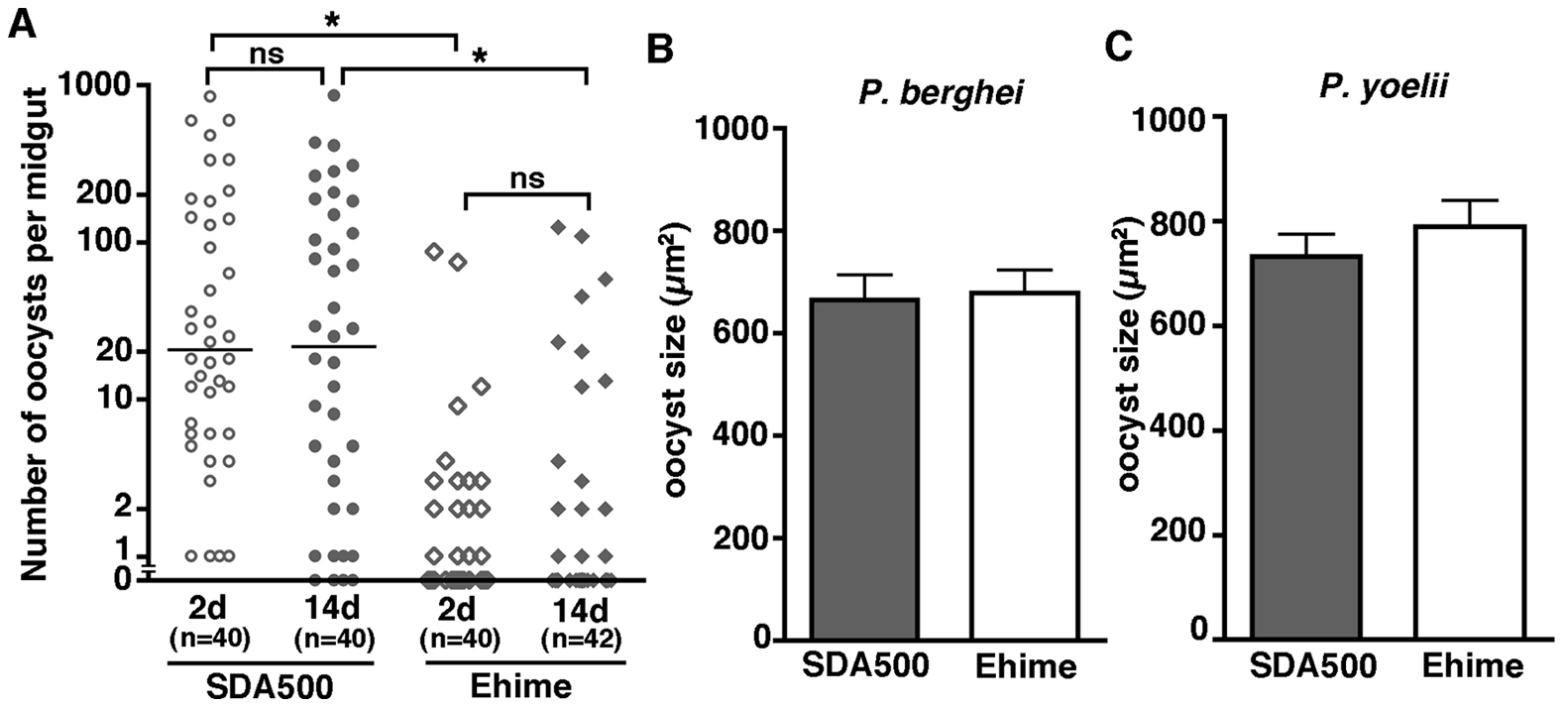
Oocyst maturation occurs normally in the midgut of the Ehime strain. (A) Persistence of malaria oocysts on the basal lamina of SDA500 and Ehime mosquitoes. SDA500 and Ehime mosquitoes were infected with Pb-GFP parasites, and dissected after parasitic transformation to oocysts from traversed ookinetes 2 d after feeding (2d) and after maturation of oocysts 14 d after feeding (14d). All dissected mosquitoes were blood-fed using the same mouse. The total number of blood-fed mosquitoes is indicated under each group’s name. The number of oocysts in each midgut was counted. The dots represent the number of oocysts present on individual midguts and the median number of oocysts is indicated by the horizontal line. *p<0.0001 (Mann-Whitney test). ns: not significant. Infection phenotypes were confirmed in three independent experiments using a different infected mouse. (B and C) Estimated size of mature oocysts in SDA500 and Ehime mosquitoes infected with *P. berghei* (B) and *P. yoelii* (C). (B) GFP-expressing area (µm^2^) was calculated. (C) DsRed-expressing area (µm^2^) was calculated. The average size of 20 oocysts is represented. Error bars represent standard deviation of three independent assays using a different infected mouse. Not significant, for Pb-oocyst size in SDA500 *vs*. Ehime (Unpaired t-test). Not significant, for Py-oocyst size in SDA500 *vs*. Ehime (Unpaired t-test).

To determine whether later stage development of the parasite is impaired in the Ehime strain, we examined oocyst maturation after blood-feeding from *P. berghei-*infected mice. Although the number of early oocysts of *P. berghei* was lower in Ehime mosquitoes than in SDA500 mosquitoes, the number of early oocysts was similar to the number of mature oocysts in both Ehime and SDA500 mosquitoes (Figure 5A and Figure S8). In SDA500 mosquitoes, the prevalence of oocysts slightly decreased during maturation from 100% to 90% (p<0.05, chi-square analysis), but not in Ehime mosquitoes (from 45% to 40%; p = 0.68, chi-square analysis). These results indicate that parasites were not lost during maturation in Ehime mosquitoes. The size of mature *P. berghei* oocysts was comparable in both mosquito strains (Figure 5B). The size of mature *P. yoelii* oocysts was also similar in both strains (Figure 5C). These results indicate that oocysts develop normally in the *Plasmodium*-refractory Ehime strain. The results also suggest that malaria oocysts in *A. stephens*i that are protected by the surrounding plasmalemma are robust, even though humoral immune molecules are abundant in mosquito hemolymph [27], [28].

## Discussion

Vectorial capacity describes the transmission of malaria parasites that target anopheline mosquitoes [29]. The results of the present study indicate that the Ehime strain of the malaria vector *A. stephensi* has a phenotype that results in reduced susceptibility to *P. berghei* and *P. yoelii* infection. In this mosquito strain, ookinete-stage parasites are impaired in the lumen and epithelium of the mosquito midgut, resulting in a decreased number of developed oocysts. In past studies, variation in *A. stephensi* susceptibility to *P. falciparum* was determined based on the assessment of oocyst number alone. However, the parasite stages that are eliminated and the mosquito factors that are involved in susceptibility are not well understood [16], [17], [30]. Gene knockdowns that increase or decrease the susceptibility of mosquitoes to rodent malaria frequently result in a similar increase or decrease in susceptibility to infection by human malaria [31]–[33]. Therefore, the susceptibility of the Ehime strain to human malaria parasites would be an interesting subject for study. Further studies that identify the molecules responsible for the variation in *Plasmodium* susceptibility using this newly identified refractory strain of *A. stephensi* would help to clarify the aspects of parasite-vector interaction that determine vectorial capacity.

*A. stephensi* is classified into 3 biological forms–type, intermediate, and *mysorensis*–based on differences in egg morphological characters that result from a polygenic system. However, the distribution of these biological forms and their roles in malaria transmission vary among endemic areas [15]. The egg morphology of the Ehime strain exhibits the properties of the *mysorensis* form. This form inhabits rural areas and is considered a poor malaria vector, whereas SDA500, a *Plasmodium*-susceptible line is of the intermediate form, which often inhabits semi-urban areas (Table 1) [15]. It would be interesting to examine the relationship between the area inhabited by these mosquitoes and malaria susceptibility, although in this regard, further field-based studies are needed.

A comparative study of vectorial capacity using *A. gambiae* revealed that the refractory strain L3–5 completely aborts malaria parasites through melanotic encapsulation of invading ookinetes. In this strain, however, there is no effect on parasitic development in the midgut lumen [1]. Binding of thioester-containing protein 1 (TEP1), a complement-like factor in mosquitoes, kills parasites and its genetic diversity controls whether ookinete encapsulation occurs [4], [25]. Remarkably, Ehime mosquitoes suppress the formation of *P. berghei* ookinetes in the midgut lumen (Figure 3B), whereas TEP1 binding, followed by epithelial nitration, occurs after midgut invasion in *A. gambiae* [34]. It therefore appears that a TEP1-independent elimination system is affecting luminal zygotes/ookinetes in Ehime mosquitoes.

Our interesting observation is that refractory Ehime mosquitoes suppress the development of both of rodent malaria parasites, *P. berghei* and *P. yoelii*. Although *P. yoelii* oocysts develops earlier than those of *P. berghei*, the maturation was not affected by the refractoriness in Ehime mosquitoes. Decreased ookinetes formation in both species imply that an acute response to feeding of a blood meal might be working in order to kill midgut stage parasites before invasion at a very early timing post blood feeding. While host blood feeding with or without parasites significantly changes the transcriptional levels of many genes expressed in the mosquito, including digestive enzymes, peritrophic matrix proteins, and innate immune factors, no direct evidence of parasite-killing factors secreted by the mosquito midgut has yet been obtained [31], [35]–[38]. Aminopeptidase activity has been amplified in *A. stephensi* Pb3–9A during selection for its refractoriness to *P. falciparum* transmission [39]. The post-feeding kinetics of digestive enzyme activity could be a candidate system to confer the refractoriness because the controlled balance of midgut proteases affect parasite development and invasion success at a number of levels [40]. Moreover, the target of unknown mosquito effectors are assumed to be the exposed surface proteins (*e.g.,* P25 and P28), because these surface antigens are well conserved in *Plasmodium* species and are the same throughout development from zygotes to ookinetes [41]. This assumption is supported by the refractoriness of Ehime mosquitoes to two rodent parasites. A species of *Enterobacter* isolated from a wild mosquito suppresses ookinete formation via production of reactive oxygen species (ROS), independently of innate mosquito immunity [42]. Therefore, the parasite defects observed in the Ehime midgut may involve colonizing of a spectrum of luminal bacteria. Further investigation of Ehime *Plasmodium*-refractory mosquitoes will provide important mechanistic insights into the parasite-vector interaction in the midgut lumen and epithelium where invasive ookinetes are impaired.

## Materials and Methods

### Ethics Statement

All animal experimental protocols were approved by the Institutional Animal Care and Use Committee of Ehime University, and the experiments were conducted according to the Ethical Guidelines for Animal Experiments of Ehime University.

### Mosquito Rearing and Parasite Maintenance

*A. stephensi* were reared at 24°C under 12 h/12 h light/dark cycle and maintained using 5% sucrose. The Ehime strain was originally provided by Dr. Masamichi Aikawa and had been reared at Ehime University for over 15 years. The original colony of Ehime was maintained at Case Western Reserve University. SDA500 and SDA500’ were kindly provided by Dr. Meiji Arai and Dr. Masao Yuda, respectively. The two SDA500 strains, derived from Imperial College London, were separately reared for over 10 years in a different laboratory. For genetic crossing, virgin females of the Ehime strain were collected within 3 h of eclosion. These females were crossed with SDA500 males, and the F_1_ progeny were used for infection experiments.

### Parasite Infection of Mosquitoes

The *P. berghei* ANKA strain expressing GFP driven by heat shock protein 70 promoter (Pb-GFP) was kindly provided by Dr. Masao Yuda [43] and was used for all *P. berghei* infections. The *P. yoelii* 17XNL strain expressing DsRed driven by elongation factor 1 α promoter (Py-RFP) was a kind gift from Dr. Stefan H. Kappe [44] and was used for all *P. yoelii* infections. Female BALB/c mice (CLEA Japan) that were 6–8 weeks of age were infected with malaria parasites via intraperitoneal injection of infected blood. For *P. yoelii* infection, phenyl-hydrazine was intraperitoneally injected to cause anemia 3 days before parasite injection. Asexual growth of parasites was monitored using Giemsa-stained smears. Six-day-old female *A. stephensi* were blood-fed on anesthetized infected mice when parasitemias were between 1–5% with mature gametocytes. Fully engorged mosquitoes were collected. Mosquitoes were maintained at 20°C during feeding for *P. berghei* infection, and 24°C for *P. yoelii* infection. For strain comparisons, all strains were blood-fed using the same mouse. Ookinete feeding of *P. berghei* was performed as follows: ookinetes of *P. berghei* were cultured as described previously with some modification [45]. Stored infected blood was injected intraperitoneally into 6–8-week-old ICR mice (CLEA, Japan) that were made anemic by phenyl-hydrazine treatment. After approximately 5 d, infected blood was collected from mice and diluted 10-fold with incomplete culture medium (RPMI1640 medium; Gibco, USA) containing 50 mg/L hypoxanthine, 25 mM HEPES, 24 mM NaHCO_3_, 50 U/ml penicillin, and 50 µg/ml streptomycin and supplemented with 20% FCS. Diluted blood samples were adjusted to pH 8.3 and incubated at 20°C for 18 h. After checking ookinete formation by Giemsa staining, cultured blood was reconstituted to 50% hematocrit with incomplete culture medium (pH 8.3). Female mosquitoes that were 6 d of age were fed the reconstituted blood using a warming glass mosquito feeder (Chemglass, NIH-0511-041JS).

### Genomic PCR for ITS2 Region

Three adult female mosquitoes were homogenized in 200 µl lysis buffer (0.1 M Tris-Hcl, pH 8.0; 0.1 M EDTA; 0.1 M NaCl; 0.5% SDS), and the homogenates were boiled at 70°C for 30 min. Potassium acetate (5 M, 44.8 µl) was added, and the homogenates were incubated for 30 min on ice. After centrifugation, the supernatant was precipitated using isopropanol and then dissolved in TE (10 mM Tris-HCl, pH 8.0; 1 mM EDTA). The rDNA ITS2 regions were amplified by PCR using primers based on conserved sequences of the 5.8S and 28S coding regions [20], [46]. The primer sequences were as follows: 5.8S primer 5′-TGTGAACTGCAGGACACATGAAC-3′ and 28S primer 5′-GGGGTAGTCACACATCACTTGAGG-3′. Sequencing analysis for the purified PCR products was performed using an ABI3100 genetic analyzer (Applied Biosystems, USA).

### Egg Morphological Characteristics

Ten blood-fed SDA500 and Ehime females were transferred to a cup lined with wet filter paper and allowed to lay eggs. The number of ridges on 50 eggs from each strain was counted under a light microscope. Eggs with 10–15, 15–17, and 17–22 ridges were designated respectively as *mysorensis*, intermediate, and type forms, based on the criteria described previously [10], [15].

### Fluorescence Imaging

To evaluate oocyst formation *in vivo*, infected mosquitoes were dissected at represented days post-blood-feeding and the midgut was isolated. Immediately after dissection, isolated midguts were placed on glass slides. For observation of early *P. berghei* oocysts at 2 days post blood-feeding, ingested blood was removed using sharp forceps. Fluorescent parasites (*P. berghei*: GFP, *P. yoelii*: DsRed) were counted using an Axio observer (Zeiss, Germany) with epi-fluorescence. The rank of sum (Mann-Whitney) test was used to determine the statistical significance of differences between mosquito strains in terms of the number of oocysts. Chi-square analysis was used to determine the statistical significance of differences between mosquito strains in oocyst prevalence. The size of *P. berghei* oocysts was measured by calculating the GFP area (µm^2^) at 12 days post blood-feeding. The size of *P. yoelii* oocysts was measured by calculating the DsRed area (µm^2^) at 8 days post blood-feeding. *P. berghei* ookinete invasion and newly developed oocysts were evaluated by dissecting infected mosquitoes at 21 and 48 h after mosquitoes fed on ookinetes The blood-containing midguts of the dissected mosquitoes were isolated and the ingested blood was removed using sharp forceps. The midguts were then fixed by placing them in 4% ice-cold paraformaldehyde for 1 hour. After washing with PBT (1× PBS containing 0.1% Triton-X100) overnight and counterstaining nuclei with 4′,6-diamidino-2-phenylindole (DAPI), the isolated midguts were placed on glass slides. The GFP-expressing parasites were counted in projection images obtained using an LSM710 confocal microscope (Zeiss, Germany). The Mann-Whitney test was used to determine the statistical significance of differences between mosquito strains in terms of the number of parasites. Image processing was performed using ImageJ (NIH, USA) and Imaris software (Bitplane, Switzerland).

### Immunocytochemistry

Preparation of samples for immunofluorescence microscopy of malaria parasites within blood meals was carried out as previously described with substantial modifications [47]. The midguts, including the entire blood meal contents, were individually homogenized and diluted in 20 µL of PBS, at 10–15 min after blood feeding for male gametes, at 16 h for *P. yoelii* ookinetes, or at 18 h for *P. berghei* ookinetes. Aliquots (1 µL) were then spotted onto a 10-well glass slide. Sample slides were air-dried and fixed in 4% paraformaldehyde for 1 h (GFP analysis) or ice-cold acetone for 2 min (all other analyses). Slides were then blocked in 5% goat serum for 30 min, and incubated with primary antibodies overnight at 4°C. After 3 PBS washes, sample slides were incubated with secondary antibodies for 2 h. After 3 further washes with PBS, nuclei were stained with DAPI. Sample slides were analyzed under an Axio observer (Zeiss, Germany) with epi-fluorescence. The total number of parasites in each sample was counted. Average values for the density of each malaria parasite stage present within each midgut examined were calculated. The Mann-Whitney test was used to determine the statistical significance of differences between mosquito strains in terms of the number of ookinetes. The following antibodies were used for immunostaining: mouse anti-Pys25 monoclonal antibody (1∶2000, [24]), mouse anti-alpha-tubulin II antiserum (1∶500, *See Antibody production*), goat anti-mouse IgG-Alexa 488 (1∶200, Invitrogen), goat anti-mouse IgG-Alexa 568 (1∶200, Invitrogen).

### Antibody Production

The C-terminal region of *P. falciparum* alpha-tubulin II (amino acid residues 156R-450E of PFD1050w) was produced using a wheat germ cell-free expression system, as previously described [48]. To create an antibody against *P. falciparum* alpha-tubulin II (anti-TubII), 2 BALB/c mice were immunized subcutaneously with 30 µg purified proteins per mouse with Freund’s complete adjuvant, followed by 30 µg purified proteins with Freund’s incomplete adjuvant. All mice received a total of three immunizations, each 3 weeks apart. The antisera were collected 14 days after the last immunization. Cross-reaction against a *P. berghei* and *P. yoelii* orthologue was confirmed (Figure 2A).

### Statistical Analysis and Experimental Description

The median values of oocyst and ookinete distributions were compared using the nonparametric Mann-Whitney test. Chi-square analysis was used to compare oocyst prevalence in different experimental groups. Unpaired t-test was used to compare exflagellation rate of male gametes and oocyst size in different experimental groups. p<0.05 was considered statistically significant. Frequency distribution of oocysts and ookinetes in each figure was a representative example. Each experiment in this study was performed as two or more independent replicates. At least 25 midguts were dissected in each experimental group of infected mosquitoes except the ookinete-feeding experiments.

## Supporting Information

Figure S1 **Sequence alignment of ITS2 in each strain of** ***A. stephens*** **i.** Nucleotides 143–608 in AY157316 are represented as the ITS2 reference sequence of *A. stephensi*.
(TIF)

Figure S2 **Other examples of the refractoriness to** ***P. berghei*** **infection in Ehime mosquitoes.** Persistence of malaria oocysts on the basal lamina of 2 strains of SDA500 and Ehime mosquitoes infected with Pb-GFP. The number of oocysts in each midgut was counted after dissection. All dissected mosquitoes were blood-fed using the same mouse in each experiment. The dots represent the number of oocysts present on individual midguts and the median number of oocysts is indicated by the horizontal line. The total number of mosquitoes blood-fed is indicated under each group’s name. *p<0.0001, **p<0.01 (Mann-Whitney test), ns: not significant.
(TIF)

Figure S3 **Other examples of the refractoriness to** ***P. yoelii*** **infection in Ehime mosquitoes.** Persistence of malaria oocysts on the basal lamina of 2 strains of SDA500 and Ehime mosquitoes infected with Py-RFP. The number of oocysts in each midgut was counted after dissection. All dissected mosquitoes were blood-fed using the same mouse in each experiment. The dots represent the number of oocysts present on individual midguts and the median number of oocysts is indicated by the horizontal line. The total number of mosquitoes blood-fed is indicated under each group’s name. *p<0.01, **p<0.05 (Mann-Whitney test), ns: not significant.
(TIF)

Figure S4 **Other examples of the intermediate phenotype of F_1_ progeny.** Persistence of malaria oocysts on the basal lamina of F_1_ progeny from a reciprocal cross of Ehime and SDA500 mosquitoes and their parental lines. The number of *P. berghei* oocysts in each midgut was counted after dissection. All dissected mosquitoes were blood-fed using the same mouse in each experiment. The dots represent the number of oocysts present on individual midguts and the median number of oocysts is indicated by the horizontal line. The total number of mosquitoes blood-fed is indicated under each group’s name. *p<0.001, **p<0.01, ***p<0.05 (Mann-Whitney test).
(TIF)

Figure S5 **Other examples of the refractoriness that suppress ookinetes formation in the midgut of Ehime.** (A and B) Frequency distribution of degenerated parasites and ookinetes in the midgut lumen of SDA500 and Ehime strains with *P. berghei* infection (A) or *P. yoelii* infection (B). Ehime and SDA500 mosquitoes were infected with Pb-GFP or Py-RFP and dissected 18 h after feeding. Spotted blood meals were stained with anti-Pys25, and the number of spherical parasites (white) and ookinetes (gray) was estimated. Error bars represent standard deviation; n = 20. p<0.001, for Pb-ookinetes in SDA500 *vs.* Ehime (Mann-Whitney test), p<0.001, for Py-ookinetes in SDA500 *vs.* Ehime (Mann-Whitney test). (C) Frequency distribution of ookinetes in the midgut lumen of SDA500 and Ehime strains with *P. berghei* infection. Error bars represent standard deviation; n = 20. p<0.001, for SDA500 *vs.* Ehime (Mann-Whitney test)
(TIF)

Figure S6 **Other examples of the refractoriness that suppress oocyst formation after feeding on ookinetes in Ehime mosquitoes.** Persistence of malaria oocysts in the basal lamina of SDA500 and Ehime mosquitoes after feeding on ookinetes. SDA500 and Ehime mosquitoes were fed cultivated Pb-GFP ookinetes and dissected 5 d after feeding. The number of oocysts in each midgut was counted. The total number of mosquitoes blood-fed is indicated under each group’s name. The dots represent the number of oocysts present on individual midguts and the median number of oocysts is indicated by the horizontal line. Two independent results were represented. *p<0.0001 (Mann-Whitney test).
(TIF)

Figure S7 **Another example of the refractoriness that suppress ookinetes traversal in Ehime mosquitoes.** Persistence of malaria parasites in the midgut of SDA500 and Ehime after feeding on ookinetes. Infected midguts were dissected at 21 and 48 h post feeding (hpf). The number at 21 hpf includes both invading ookinetes and young oocysts, and the number at 48 hpf indicates early oocysts. The total number of mosquitoes blood-fed is indicated under each group’s name. The dots represent the number of oocysts present on individual midguts and the median number of oocysts is indicated by the horizontal line. *p<0.01 (Mann-Whitney test).
(TIF)

Figure S8 **Other examples of the normal maturation of** ***P. berghei*** **oocysts in Ehime mosquitoes.** (A) Persistence of early oocysts at 2 d after feeding (2d) and mature oocysts at 14 d after feeding (14d). (B) Persistence of early oocysts at 2 d after feeding. SDA500 and Ehime mosquitoes were infected with Pb-GFP parasites and dissected at represented day. All dissected mosquitoes were blood-fed using the same mouse in each experiment. The total number of mosquitoes blood-fed is indicated under each group’s name. The number of oocysts in each midgut was counted. The dots represent the number of oocysts present on individual midguts and the median number of oocysts is indicated by the horizontal line. *p<0.01, **p<0.0001 (Mann-Whitney test). ns: not significant.
(TIF)

Table S1 **Decreased prevalence of** ***P. berghei*** **and** ***P. yoelii*** **oocysts in Ehime** ***A. stephensi*** **.** Distributions of oocysts number are represented in Figures S2–S4.
(DOCX)
