# Supplementary figures and images for: Phenotypic Dissection of a Plasmodium-Refractory Strain of Malaria Vector Anopheles stephensi: The Reduced Susceptibility to P. berghei and P. yoelii
Source: PLoS One. 2013 May 23;8(5):e63753. doi: 10.1371/journal.pone.0063753 (PMC3662785; doi:10.1371/journal.pone.0063753)

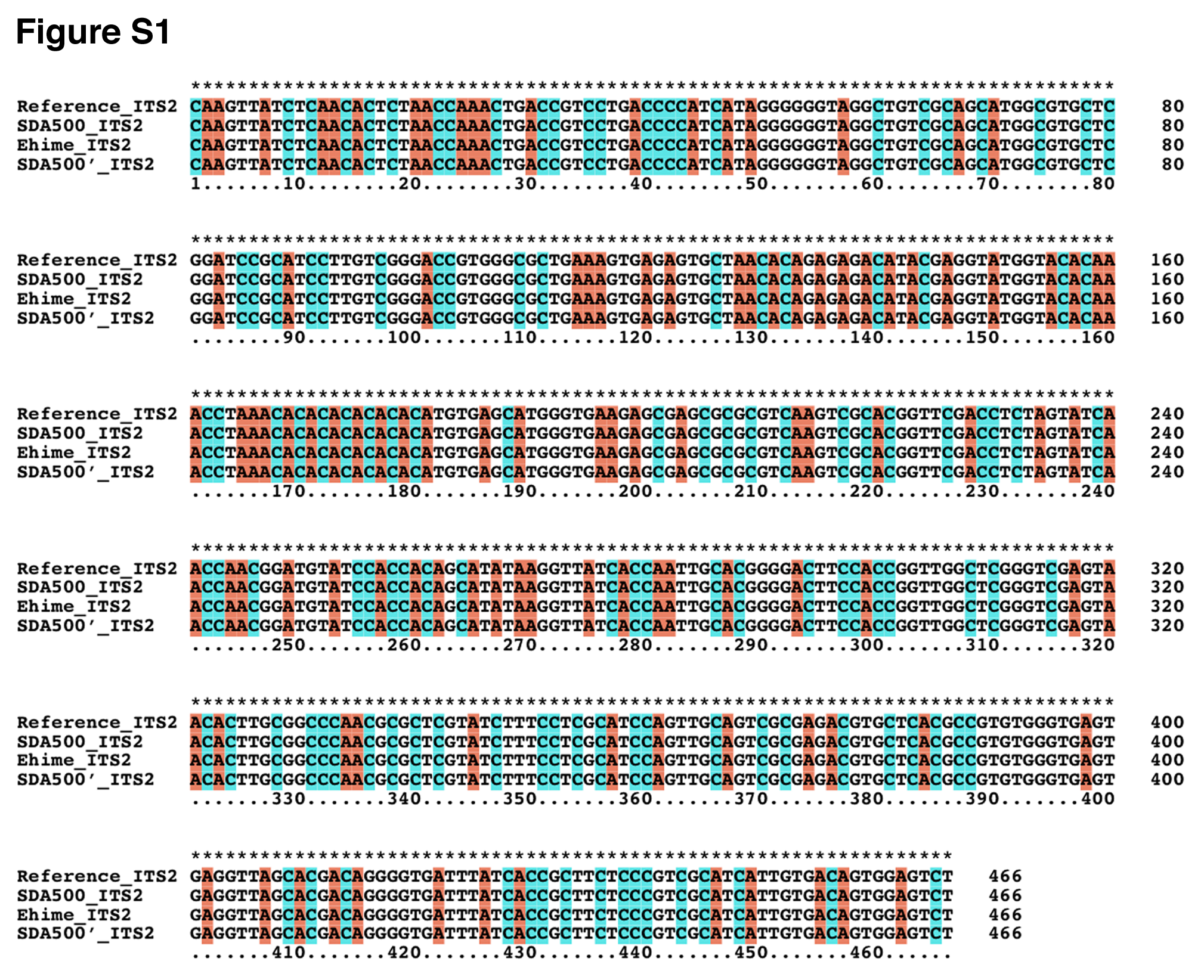

Supplement: Figure S1 — Sequence alignment of ITS2 in each strain of A. stephens i. Nucleotides 143–608 in AY157316 are represented as the ITS2 reference sequence of A. stephensi. (TIF) [file pone.0063753.s001.tif]

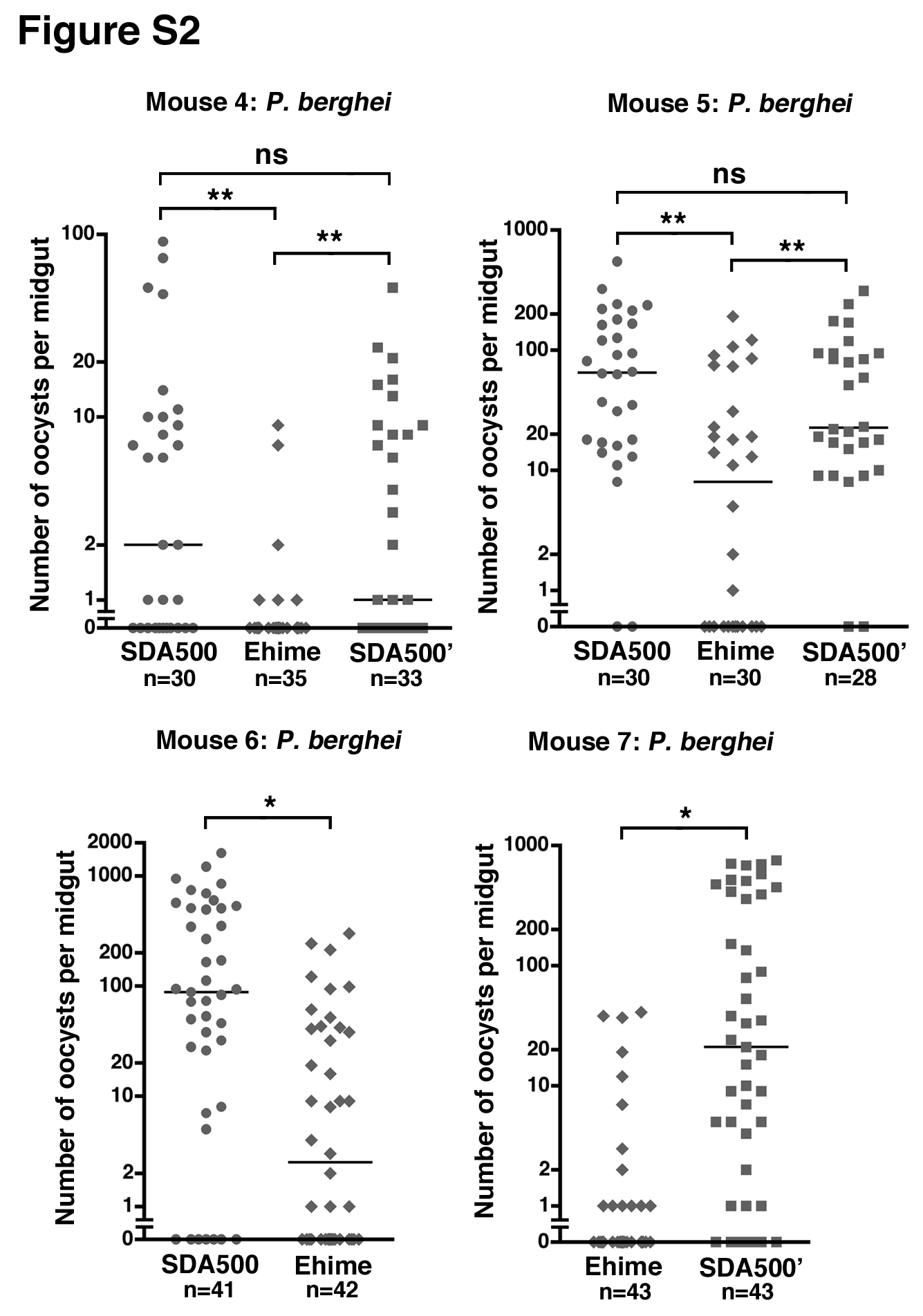

Supplement: Figure S2 — Other examples of the refractoriness to P. berghei infection in Ehime mosquitoes. Persistence of malaria oocysts on the basal lamina of 2 strains of SDA500 and Ehime mosquitoes infected with Pb-GFP. The number of oocysts in each midgut was counted after dissection. All dissected mosquitoes were blood-fed using the same mouse in each experiment. The dots represent the number of oocysts present on individual midguts and the median number of oocysts is indicated by the horizontal line. The total number of mosquitoes blood-fed is indicated under each group’s name. *p<0.0001, **p<0.01 (Mann-Whitney test), ns: not significant. (TIF) [file pone.0063753.s002.tif]

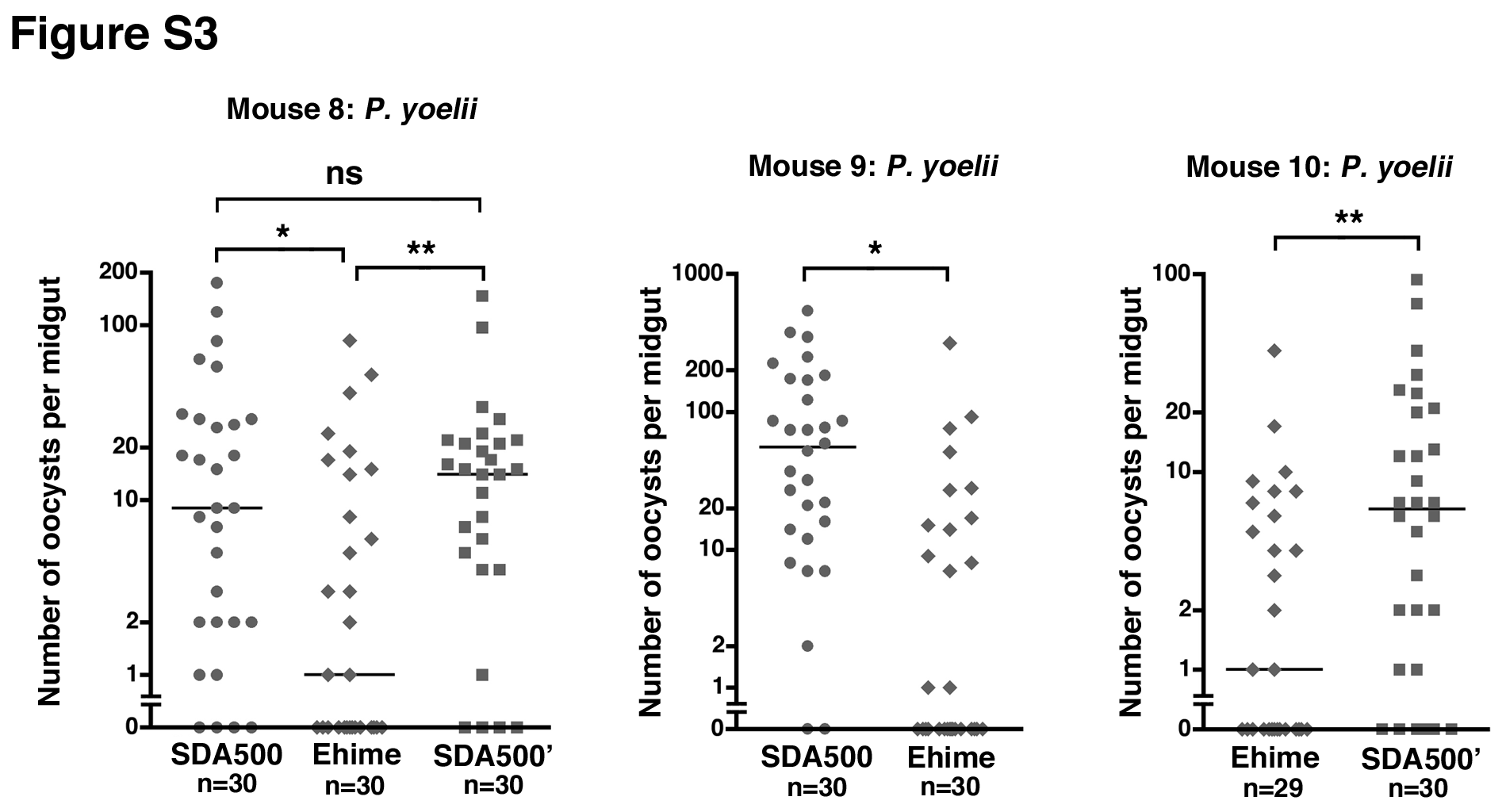

Supplement: Figure S3 — Other examples of the refractoriness to P. yoelii infection in Ehime mosquitoes. Persistence of malaria oocysts on the basal lamina of 2 strains of SDA500 and Ehime mosquitoes infected with Py-RFP. The number of oocysts in each midgut was counted after dissection. All dissected mosquitoes were blood-fed using the same mouse in each experiment. The dots represent the number of oocysts present on individual midguts and the median number of oocysts is indicated by the horizontal line. The total number of mosquitoes blood-fed is indicated under each group’s name. *p<0.01, **p<0.05 (Mann-Whitney test), ns: not significant. (TIF) [file pone.0063753.s003.tif]

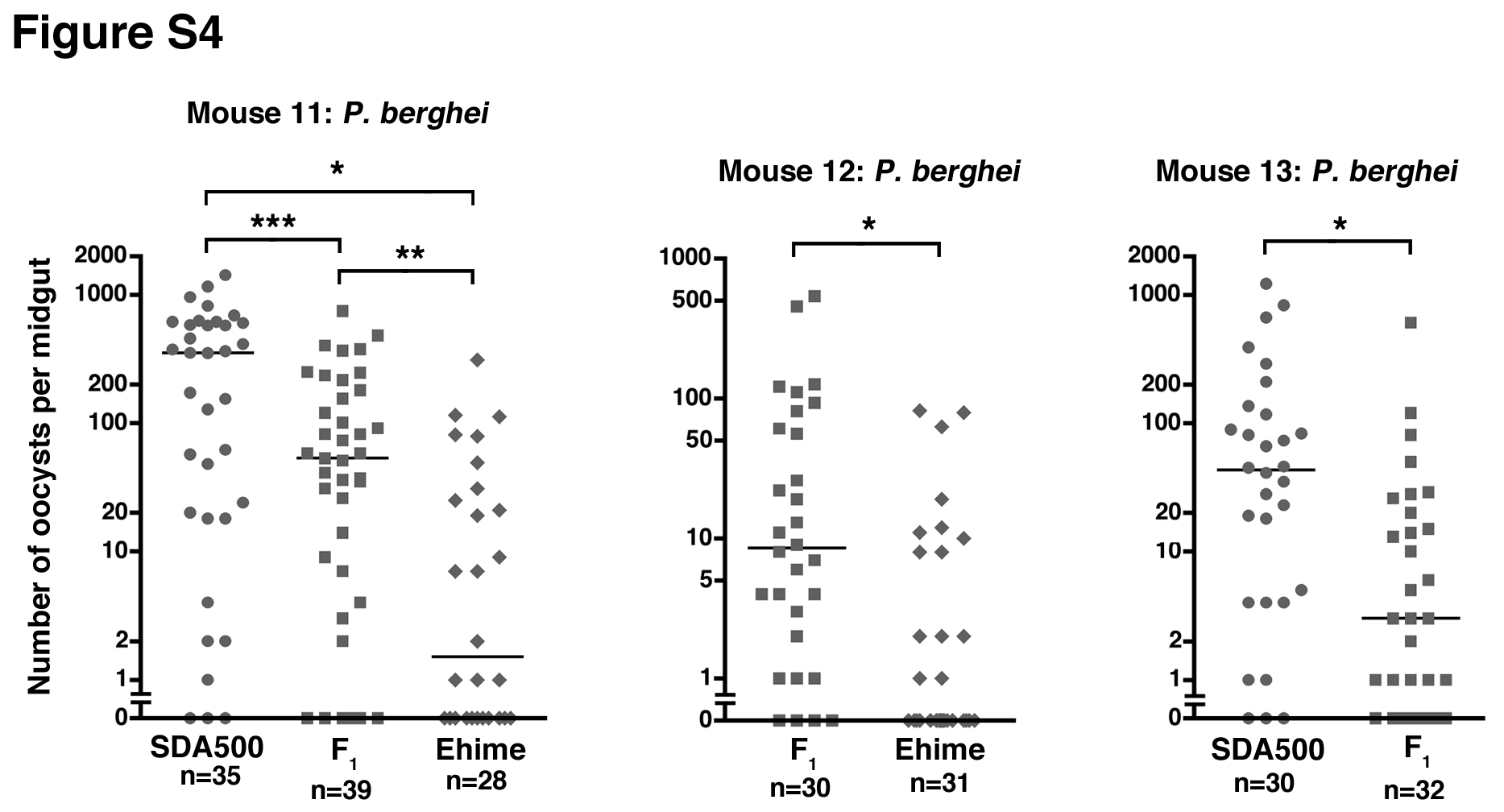

Supplement: Figure S4 — Other examples of the intermediate phenotype of F1 progeny. Persistence of malaria oocysts on the basal lamina of F1 progeny from a reciprocal cross of Ehime and SDA500 mosquitoes and their parental lines. The number of P. berghei oocysts in each midgut was counted after dissection. All dissected mosquitoes were blood-fed using the same mouse in each experiment. The dots represent the number of oocysts present on individual midguts and the median number of oocysts is indicated by the horizontal line. The total number of mosquitoes blood-fed is indicated under each group’s name. *p<0.001, **p<0.01, ***p<0.05 (Mann-Whitney test). (TIF) [file pone.0063753.s004.tif]

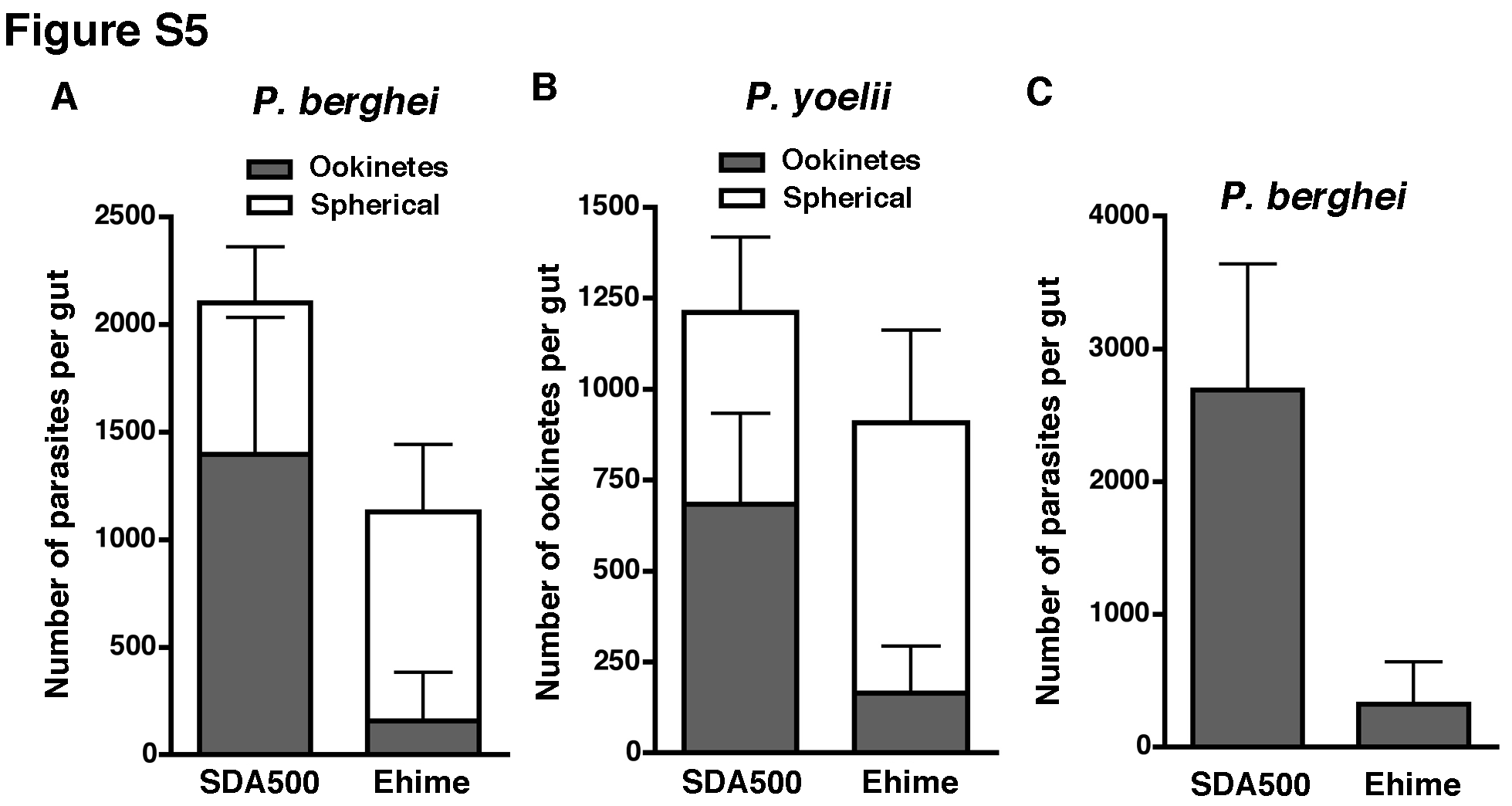

Supplement: Figure S5 — Other examples of the refractoriness that suppress ookinetes formation in the midgut of Ehime. (A and B) Frequency distribution of degenerated parasites and ookinetes in the midgut lumen of SDA500 and Ehime strains with P. berghei infection (A) or P. yoelii infection (B). Ehime and SDA500 mosquitoes were infected with Pb-GFP or Py-RFP and dissected 18 h after feeding. Spotted blood meals were stained with anti-Pys25, and the number of spherical parasites (white) and ookinetes (gray) was estimated. Error bars represent standard deviation; n = 20. p<0.001, for Pb-ookinetes in SDA500 vs. Ehime (Mann-Whitney test), p<0.001, for Py-ookinetes in SDA500 vs. Ehime (Mann-Whitney test). (C) Frequency distribution of ookinetes in the midgut lumen of SDA500 and Ehime strains with P. berghei infection. Error bars represent standard deviation; n = 20. p<0.001, for SDA500 vs. Ehime (Mann-Whitney test) (TIF) [file pone.0063753.s005.tif]

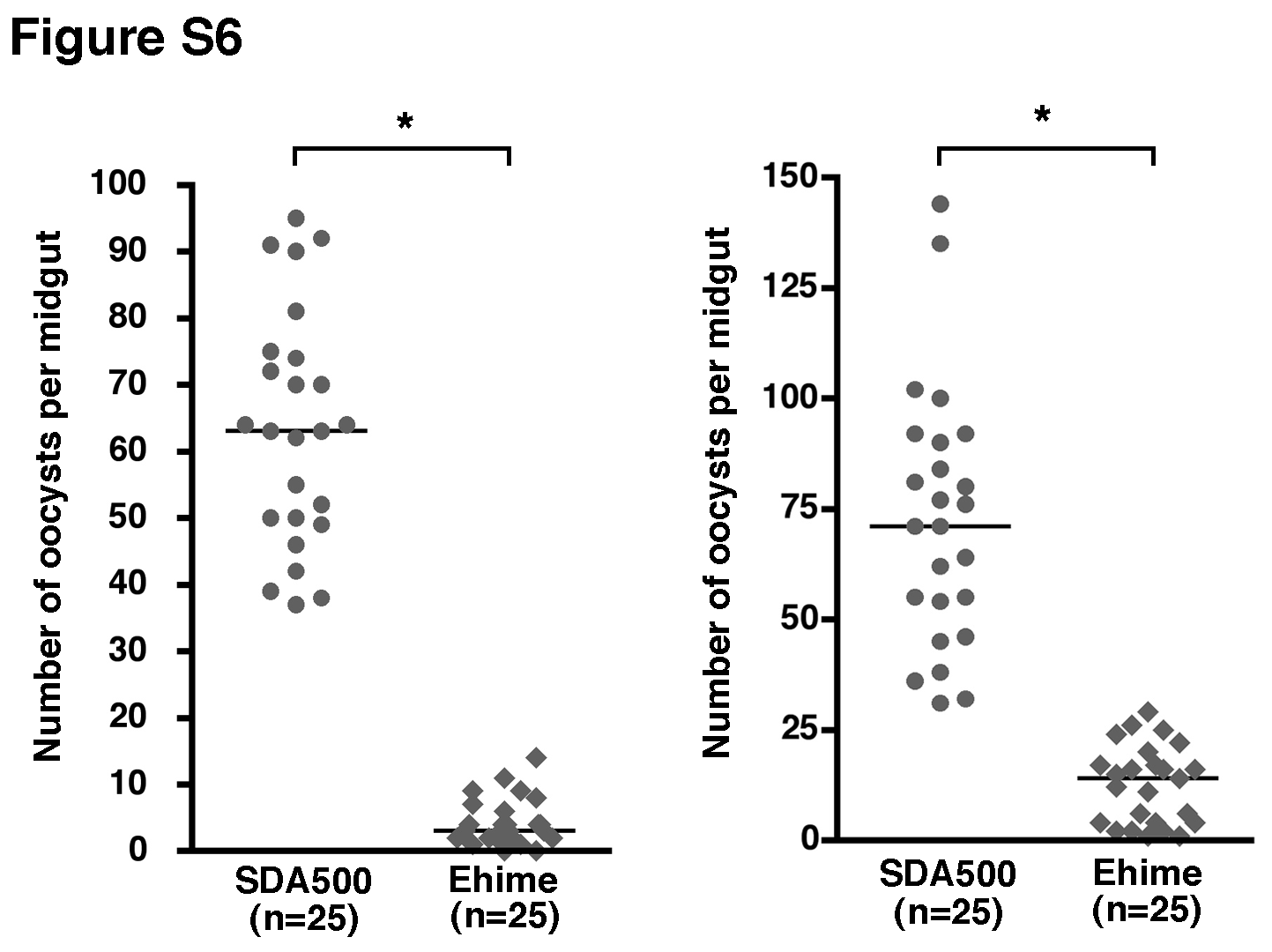

Supplement: Figure S6 — Other examples of the refractoriness that suppress oocyst formation after feeding on ookinetes in Ehime mosquitoes. Persistence of malaria oocysts in the basal lamina of SDA500 and Ehime mosquitoes after feeding on ookinetes. SDA500 and Ehime mosquitoes were fed cultivated Pb-GFP ookinetes and dissected 5 d after feeding. The number of oocysts in each midgut was counted. The total number of mosquitoes blood-fed is indicated under each group’s name. The dots represent the number of oocysts present on individual midguts and the median number of oocysts is indicated by the horizontal line. Two independent results were represented. *p<0.0001 (Mann-Whitney test). (TIF) [file pone.0063753.s006.tif]

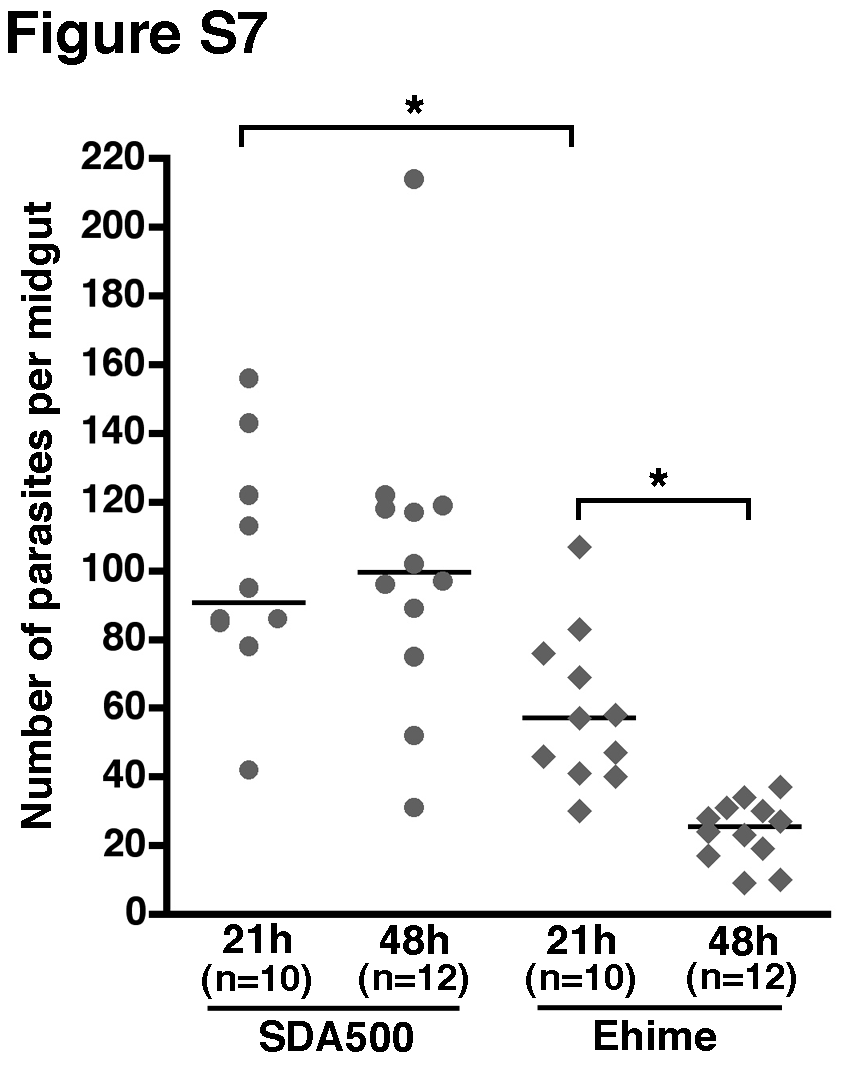

Supplement: Figure S7 — Another example of the refractoriness that suppress ookinetes traversal in Ehime mosquitoes. Persistence of malaria parasites in the midgut of SDA500 and Ehime after feeding on ookinetes. Infected midguts were dissected at 21 and 48 h post feeding (hpf). The number at 21 hpf includes both invading ookinetes and young oocysts, and the number at 48 hpf indicates early oocysts. The total number of mosquitoes blood-fed is indicated under each group’s name. The dots represent the number of oocysts present on individual midguts and the median number of oocysts is indicated by the horizontal line. *p<0.01 (Mann-Whitney test). (TIF) [file pone.0063753.s007.tif]

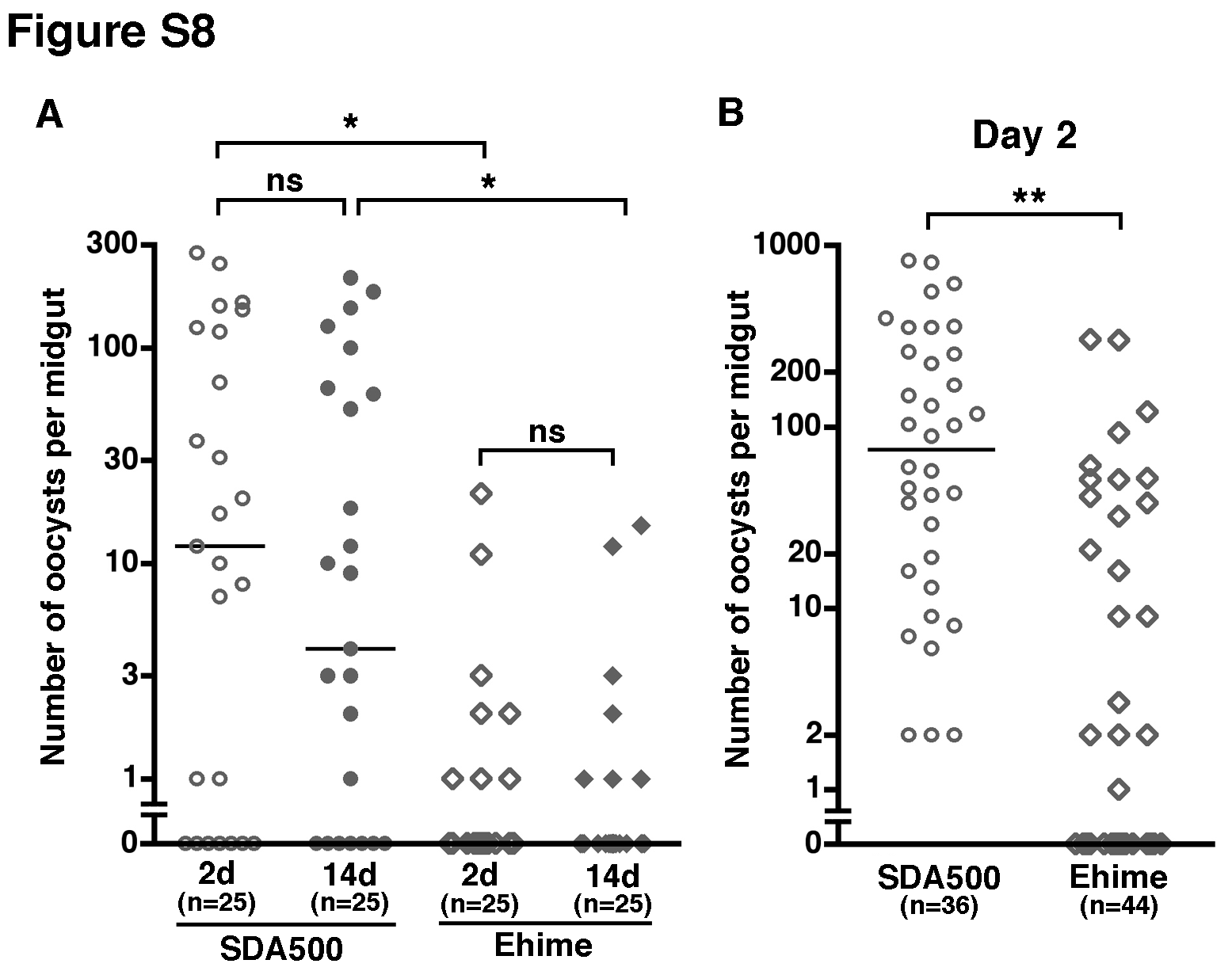

Supplement: Figure S8 — Other examples of the normal maturation of P. berghei oocysts in Ehime mosquitoes. (A) Persistence of early oocysts at 2 d after feeding (2d) and mature oocysts at 14 d after feeding (14d). (B) Persistence of early oocysts at 2 d after feeding. SDA500 and Ehime mosquitoes were infected with Pb-GFP parasites and dissected at represented day. All dissected mosquitoes were blood-fed using the same mouse in each experiment. The total number of mosquitoes blood-fed is indicated under each group’s name. The number of oocysts in each midgut was counted. The dots represent the number of oocysts present on individual midguts and the median number of oocysts is indicated by the horizontal line. *p<0.01, **p<0.0001 (Mann-Whitney test). ns: not significant. (TIF) [file pone.0063753.s008.tif]
