# Supplementary material for: Phenotypic Dissection of a Plasmodium-Refractory Strain of Malaria Vector Anopheles stephensi: The Reduced Susceptibility to P. berghei and P. yoelii
Source: PLoS One. 2013 May 23;8(5):e63753. doi: 10.1371/journal.pone.0063753 (PMC3662785; doi:10.1371/journal.pone.0063753)
Supplement: Table S1 — Decreased prevalence of P. berghei and P. yoelii oocysts in Ehime A. stephensi . Distributions of oocysts number are represented in Figures S2–S4. (DOCX) [file pone.0063753.s009.docx]

Table S1

| Experiments | Parasite | Mosquito strain | n | Prevalence (%)^a^ | p-value^b^ |
| --- | --- | --- | --- | --- | --- |
| Mouse 4 | *P. berghei* | SDA500 | 30 | 63.3 | <0.001^c^ |
| (Figure S2) |  | Ehime | 35 | 17.1 | - |
|  |  | SDA500' | 33 | 54.5 | <0.001^c^ |
| Mouse 5 | *P. berghei* | SDA500 | 30 | 93.3 | <0.01^c^ |
| (Figure S2) |  | Ehime | 30 | 60.0 | - |
|  |  | SDA500' | 28 | 92.9 | <0.01^c^ |
| Mouse 6 | *P. berghei* | SDA500 | 41 | 82.9 | <0.05^c^ |
| (Figure S2) |  | Ehime | 42 | 59.5 | - |
| Mouse 7 | *P. berghei* | Ehime | 43 | 34.9 | - |
| (Figure S2) |  | SDA500' | 43 | 83.7 | <0.001^c^ |
| Mouse 8 | *P. yoelii* | SDA500 | 30 | 86.7 | <0.01^c^ |
| (Figure S3) |  | Ehime | 30 | 53.3 | - |
|  |  | SDA500' | 30 | 80.0 | <0.05^c^ |
| Mouse 9 | *P. yoelii* | SDA500 | 30 | 93.3 | <0.001^c^ |
| (Figure S3) |  | Ehime | 30 | 46.7 | - |
| Mouse 10 | *P. yoelii* | Ehime | 29 | 51.7 | - |
| (Figure S3) |  | SDA500' | 30 | 80.0 | <0.05^c^ |
| Mouse 11 | *P. berghei* | SDA500 | 35 | 91.4 | <0.01^c^ |
| (Figure S4) |  | F_1_ | 39 | 84.6 | ns^d^, <0.05^c^ |
|  |  | Ehime | 28 | 60.7 | - |
| Mouse 12 | *P. berghei* | F_1_ | 30 | 86.7 | <0.001^c^ |
| (Figure S4) |  | Ehime | 31 | 45.2 | - |
| Mouse 13 | *P. berghei* | SDA500 | 30 | 90.0 | - |
| (Figure S4) |  | F_1_ | 32 | 71.9 | ns^d^ |

^a^ The oocyst prevalence was calculated by dividing the the number of oocyst-positive mosquitoes by the total number of mosquitoes blood-fed.

^b^ p-value was calculated by chi-square analysis.

^c^ compared with oocyst prevalence in Ehime. ^d^ compared with oocyst prevalence in SDA500.
